# The Gift-Giving Craze

**Published:** 1885-01

**Authors:** Ben. H. Pratt


### THE BISTOURY.

Vol. XX. ELMIRA, N. Y., JANUARY, 1885. No. 4.

### Contributions.

### THE GIFT-GIVING CRAZE.

BEN. H. PRATT.

The giving of gifts at Christmas time has well nigh ceased to be a custom, having developed into a craze.

It is unquestionably desirable that there should be a season, at least annual in its occurrence, when friends and kinsfolk should bestow gifts, one upon the other.

Christmas is perhaps as good a time for this as any, for if there be a day upon which the whole Christian world ought to rejoice and be exceeding glad, that day is the one which commemorates the Savior's birth.

But are we not losing sight of the real intent of the commemorative time, and merging it into a general donational event? And is the spirit of all this giving at Christmas time entirely in consonance with the day upon which it occurs? Isn't the desire rather to enjoy the pleasure one gives one's friends, and to congratulate one's self upon one's array of presents, than to celebrate a Christian festival?

It is not difficult to trace the causes of this malappropriation of the Christmas time. It is a day to be glad upon, to make merry withal, and to strive that nothing of a saddening sort shall enter into its observance. From a day of gladness, there is nothing more natural than that it should become one on which that which makes glad most thoroughly should be resorted to. As there is nothing so gladdening as the giving and receiving of simple gifts, it is the most natural thing in the world that the exchange of mementoes should have become the supreme characteristic of the day.

But like every other good thing, this giving may become, from a simple observance of the custom, a craze. It has become such. It is no longer a modest interchange of glad sentiment, and a bestowal of momentoes of friendship and attachment, but a grand gala time for the indiscriminate and too often ostentatious display of one's liberality, or of one's wealth, or of one's pride. Christmas gifts are no longer confined within the range of friendships and connections. The field is broadening year by year, and instead of its remaining the beautiful custom it once was, is becoming a mere means of revealing one's extravagance, or his lavish disregard of expense, or his reckless craving for esteem or notoriety among his fellows.

To such an extent has the custom of making presents on Christmas grown, that ordinary people are either chagrinned by their inability to match their associates in their giving or are seriously injured by attempting to do more than they can afford. This latter phase of the question has become a momentous one with many. For months before the day arrives, thousands of people are actually groaning in spirit, because they know that they cannot, without cheating somebody, meet the demand upon their purses which the Christmas time is sure to bring. They remember the lavish gifts bestowed upon them at some previous occasion by this and that giver; they feel that they have been involuntarily put under special obligations to many people, and that these persons expect to be as handsomely remembered at the coming Christmas as you were remembered at the last. It is a trying ordeal to determine whether you will take the risk of attempting to reciprocate in value, or whether you will submit to being thought an ingrate.

Were this giving of presents on Christmas day limited to intimate friends and relatives, people who know precisely what one's circumstances are, and know, too, what one's sentiments are, then the bestowal would not be rated, as it most frequently is, purely for its value, but in accordance with the affection and friendship of the giver. It is not the interchange of gifts within the family circle that is burdensome, but that which is expected between those with whom one is supposed to maintain excessively cordial social relations. It is society rather than connexional relations that most severely tests one's liberality at Christmas time. The custom grows so rapidly out of all proportion to the ability of the interchangers of gifts, and each is so anxious to excel every other in his giving, that in a very few years that which was once a trifling exchange of mementoes becomes almost an endowment, and out of all proportion to the givers' means. The temptation to burden one's self, to do much more than one can afford to do, has been assisted in large measure by the dealers in holiday goods, as they are termed. The most lavish displays are made by these gift caterers, and thousands of people are employed in the one occupation, the whole year around, of devising and creating novelties to catch the eye and tempt the fancy of the holiday gift buyer.

With this temptation constantly before their eyes, the most sensible of men and women lose their heads, as the saying goes, and buy pell-mell, without any serious reflection upon the condition of their pockets or upon anything but the existence of these holiday baubles, and are unconsciously forced into the belief that their social duty lies in the direction of purchase and bestowal, whether the things purchased be appropriate to the occasion, to the giver or to the recipient. The great demand stares all in the face; you must give, give much, give to many, and give the most beautiful and costly things you can find and pay for in the tempting shops. It is no matter whether or not your friend wants what you buy for him; and the need of anything is never a consideration. Christmas gift recipients are never supposed to *need* anything. It is not "good form," says society, to give needed things, because you thus imply a certain sort of inferiority in the gifted, and that he or she could not have obtained what was wanted except through your benevolence. Benevolence must not come into the account in Christmas gift bestowing. It must be pure delightsomeness, however empty that sentiment may be, and your aim must be to tickle rather than to profit the individual you purpose to honor; or, to express it more truthfully, the person through whom you declare yourself the honorer of yourself.

The great Christmas festival of 1884 has passed. Its experiences for this year have gone into your biography. They are episodical incidents of your history. You can now look back upon the 25th day of last December and contemplate its transactions with some degree of cool-headed philosophy. Honest, now, what is your private opinion of last Christmas day, so for as it relates to yourself and those whose joys and sorrows and innermost sentiments you share to a large extent? You can readily recall just how you felt, and just how your folks, and your friends, and your friends' folks felt about it. It is all clear in your mind. The many talks over the coming time are fresh in mind. The increasing fervor over gifts, as the day drew nearer, is quite a positive recollection. You recall the few days before, how you couldn't more than half attend to your customary avocations in your bewilderment as to whether each article you had purchased was the proper thing for the parties to whom it was to be sent, and as to whether what you were going yet to provide, could be obtained, and if it could, whether there wouldn't be something else a good deal more appropriate, but which you would be very unlikely to discover before it was too late to make a change. You recall the night before, how, all but the last "things" purchased, you had to manoeuvre one way and another to keep everybody in the deepest ignorance as to what you were doing, and how exceedingly and happily distressed and excited you were. You remember all this, of course, and now, honestly ———

But again. You easily summon up memories of how you tumbled upon things, very accidently, that you were supposed to have no business with; how you saw things and heard things which you at once realized you couldn't say anything about because it would destroy the charm that was in store for the Christmas morning. Some things that were not intended for you to know at all were revealed to you by such awfully unlucky accidents, and what would not be at all surprising to you on Christmas day must seem to be the very acme of astonishment. You recall all this without effort. You may have had to lie a little, both in speech and action, to prevent a complete spoiling of perhaps months, or at least of weeks of calculation and profound exercise of ingenuity. You couldn't forget this so soon, and therefore it is a good time to ask you to say, honestly and truly, what do you think of the whole thing now? Sitting right there, with the results of last Christmas day's giving in your pockets, on your fingers, in your neck scarf, on your wrists, in your jewel case, on your cabinet, in your closet, on your shelves, about your walls, on your side board, on your mantel, all about you—say now. is one half of the things you have, useful things? Is one quarter or one eighth or one tenth of them things you really value because of the things themselves? Are you not a little disappointed, even you who received hosts of things, because the very things you wanted and were longing for are not among your treasures?

Then again—do you feel as though the people that have sent you these gifts were actuated by a pure desire to make you happy on that Christmas day, or were they merely making some helter-skelter return for favors from yourself on some previous Chritsmas, or in anticipation of receiving something from you at some coming return of the Christmas season? Have you not received some things that you don't care tuppence about; and other things that you don't know what in the world you will ever do with; and still other things which, whether useful or desirable, you have just a little wish that he or she who sent them, hadn't done so, or bothered themselves about you? Isn't there a sense of heavy obligation laid upon you by some of all the array of material that you have suddenly become the possessor of? Do you feel quite comfortable over it all, now that it is all over and done? Honest, now, do you? And then do you feel quite compensated for all the labor and time and worry and management and ingenuity and perplexity that you put into the ante Christmas work? Has it paid you to get yourself all worked up into the frenzy that you can't deny you experienced during the days or weeks before the gift date came? How about your pocket? Is your pecuniary exhaustion such as may be recovered from shortly, or will you have to peg and dig and scrimp and deny yourself for months to come because of your reckless giving and as senseless receiving? Honest, now! No prevaricating! Is the memory all joy?

How are we going to help ourselves? That's a question many are now asking within their silent selves. The trouble—if it be a trouble —is on the increase. There's only one way out of it. It is to be honest and have everybody think you are mean. It is to give, what you give, of only things useful, and thus run the chance of insulting somebody. It is to simplify your giving and lay yourself open to a very poor opinion from several people. It is to narrow down the field of your giving to such limits as your circle of intimacy and relationship circumscribe. It is to become a reformer—the very worst sort of a reformer, a social reformer, that everybody takes a shy at upon every opportunity and makes it deucidly uncomfortable for him or her—and make yourself a social target. It is to fly in the face of all the disciples of Mrs. Grundy and prove that you are as pachydermatous as a rhinoceros or an alligator. It is the only way to stop this exceedingly unchristian like way of spending Christmas. You won't do it. You will go right along and make believe that you are supremely happy in exhausting your pocket, your ingenuity, your time, your patience, your humanity and your Godliness for the sake of keeping up with the hurly-burly of the hour. There's only one other way to stop the craze, and that is to let it go on until you are clean done for, mentally, morally, physically and pecuniarily; and then you'll quit and fall out, and may be joined the next year by a few more, and then attention may be drawn to the situation and an emancipation from the craze may result. It may be some years before we can ever get back to anything like a sensible interchange of Christmas mementoes, but it must come in time, and before the millennium, for the millennial year could not tolerate such a wholesale injustice and abomination as is the Christmas gift-giving craze which now makes the social connection a grim misery for every one of its votaries.

